# Venoarterial carbon dioxide gradient utility as a criterion for blood transfusion at the intensive care unit

**DOI:** 10.1186/2197-425X-3-S1-A221

**Published:** 2015-10-01

**Authors:** JL Navarro, A Sanchez-Calzada, R Gastelum, L Delgado, O Torres, P Romano, E Monares, C Gilberto, J Franco

**Affiliations:** ABC Medical Center, Intensive Care, Mexico, Mexico; Hospital San Angel Inn Universidad, Intensive Care, Mexico, Mexico

## Intr

Currently there is controversy about criteria for blood transfusion in critically ill patients by a level of hemoglobin and specially as a strategy to raise the venous oxygen saturation (Sv02).

## Objectives

To analyze de utility of the venoarterial carbon dioxide gradient (V-a PC02) to detect those patients who will respond with a rise of Sv02 to blood transfusion.

## Methods

Patients within their first 12 hours from admission to the intensive care unit (ICU) during the hemodynamic optimization protocol in which blood transfusion was decided to rise the Sv02 after hemodynamic optimization: central venous pressure (CVP)>8 mmHg, mean arterial pressure (MAP)>65 mmHg, peripheral oxygen saturation (Sp02)>90%. Pre and post transfusion hemoglobin, venous oxygen saturation (Sv02_)_, and V-a PC02 were measured, then divided in two groups: "Responders" (R) if a rise >5%. In Sv02 after transfusion was present and in "Non responders" (NR) with a rise < 5% in Sv02 after transfusion. Receiver Operating Characteristic (ROC) curve analysis was performed to asses the utility of the pre transfusion V-a PC02 as a tool to predict responsiveness of the Sv02 to blood transfusion.

## Results

73 patients were analyzed, mean age of 68 ± 2, with 35 (47.9%) males, 25 (34,25%) patients in the R group and 48 (65,75%) in the NR group.

ROC curve analysis were performed resulting in an area under the curve of 0.82 (p < 0.01; CI 0.73-0.91) with a pre transfusion V-a PC02 cutoff value of ≥6 showing a sensibility 66% of and a specificity of 84% for predicting those patients who will not respond with a rise greater than 5% in the post transfusion Sv02.

## Conclusions

V-a PC02 >6 mmHg identifies those patients that will no show a rise of the Sv02 as a response to blood transfusion.

